# Skin-Grafting, Our Present Knowledge

**Published:** 1882-05

**Authors:** 


### SKIN GRAFTING, OUR PRESENT KNOWLEDGE.

This is well stated by Dr. Christopher Johnson (*International Surgery*), Vol. 1, p. 549.

*a.*—It affords an admirable means of accelerating and facilitating cicatrization.

*b.*—The pellicle produced by its aid is less prone to contraction, and contracts less than an ordinary cicatrix.

*c.*—The deeper layers of the epidermic elements are the chief factors of growth.

*d.*—The growing cicatrix is formed at the expense of the embryonal cells of the granulating surface, stimulated into activity by the presence of the living cells of the graft.

*e.*—This stimulus first showing energy in and about central islands of new growth, induces similar activity at the hitherto dormant margin of the ulcer.

*f.*—Grafts may retain vitality, and be effective long after separation from the body.

*g.*—Small grafts the size of a millet seed, for example, are generally preferable to larger ones, although much larger grafts have had their successes and advocates.

*h.*—Grafts should be obtained from the patient himself, if possible; but in all cases the danger of specific inoculation ought to be present in the mind of the surgeon who borrows grafts from one subject for application upon another, or who practises heteroplasty.

*i.*—Grafts furnished by the aged are less disposed to adhere than those obtained from the young, and sometimes fail entirely.

*j.*—Grafts obtained from one race of men may be successfully used on individuals of another race; and animal grafts may be transplanted upon human beings and provoke cicatrization.

*k.*—Foul surfaces, or those of persons in bad health, will refuse to accept good grafts; but with improvement or establishment of the health of the individual bearing an ulcer, and the appearance of healthy granulations, a favorable result of skin grafting may be anticipated.

*l.*—Finally, the great benefits accruing from successful skin grafting far outweigh its drawbacks, which are the pain of the operation, and unless amputated limbs be utilized, the consecutive pain in the parts yielding the grafts, whether these be autoplastic or heteroplastic.—*Detroit Lancet.*

